# From Brush to Dendritic Structure: Tool for Tunable Interfacial Compatibility between the Iron-Based Particles and Silicone Oil in Magnetorheological Fluids

**DOI:** 10.1021/acs.langmuir.3c03736

**Published:** 2024-03-02

**Authors:** Szymon Kozłowski, Josef Osička, Marketa Ilcikova, Monika Galeziewska, Miroslav Mrlik, Joanna Pietrasik

**Affiliations:** † Department of Chemistry, Institute of Polymer and Dye Technology, Lodz University of Technology, Stefanowskiego 16, 90-537 Lodz, Poland; ‡ Centre of Polymer Systems, Tomas Bata University in Zlin, University Institute, Trida T. Bati 5678, 76001Zlin,Czech Republic; § Slovak Academy of Sciences, Polymer Institute, Dubravska cesta 9, 845 41 Bratislava, Slovakia; ∥ Department of Physics and Materials Engineering, Faculty of Technology, Tomas Bata University, Vavreckova 5669, 76001Zlin,Czech Republic

## Abstract

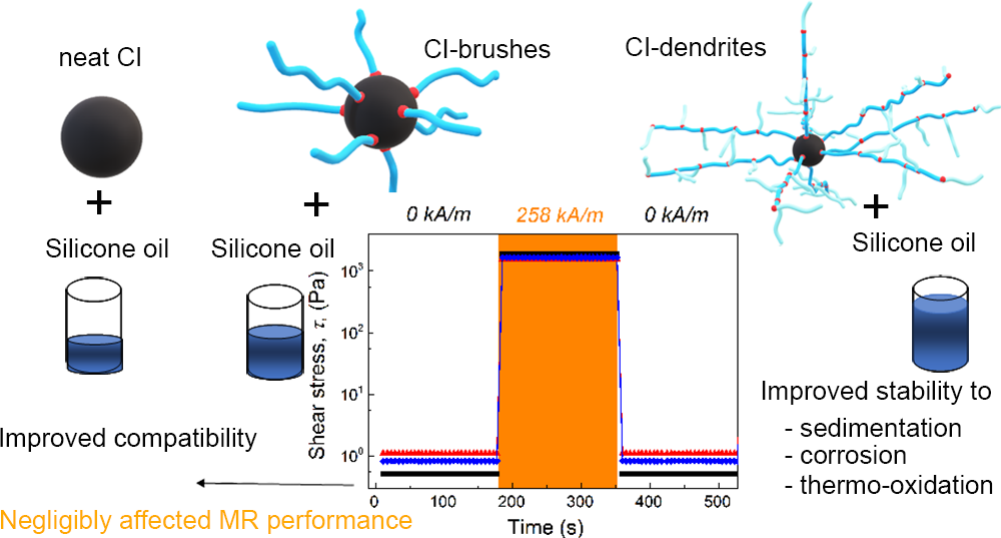

Comprehensive magnetic particle stability together with compatibility between them and liquid medium (silicone oil) is still a crucial issue in the case of magnetorheological (MR) suspensions to guarantee their overall stability and MR performance. Therefore, this study is aimed at improving the interfacial stability between the carbonyl iron (CI) particles and silicone oil. In this respect, the particles were modified with polymer brushes and dendritic structures of poly(2-(trimethylsilyloxy)ethyl methacrylate) (PHEMATMS), called CI-brushes or CI-dendrites, respectively, and their stability properties (corrosion, thermo-oxidation, and sedimentation) were compared to neat CI ones. Compatibility of the obtained particles and silicone oil was investigated using contact angle and off-state viscosity investigation. Finally, the magneto-responsive capabilities in terms of yield stress and reproducibility of the MR phenomenon were thoroughly investigated. It was found that MR suspensions based on CI-brushes had significantly improved compatibility properties than those of neat CI ones; however, the CI-dendrites-based suspension possessed the best capabilities, while the MR performance was negligibly suppressed.

## Introduction

Smart systems are usually hybrid composites or integrated systems of advanced materials^1^ that can respond to external physical or chemical stimuli in a controlled manner to perform specified tasks.^2,3^ They react to environmental changes, such as mechanical stress^4^ and strain,^5^ hydrostatic pressure,^6^ magnetic^7−10^ and electric field,^11−14^ temperature,^15−17^ light,^18−20^ pH,^21^ and moisture,^22^ and then come back to the original state after removing the stimuli.^2,3^ External stimuli can cause different responses such as changes in size, color, moisture, and viscosity of flow.^3^ Smart materials, due to their intelligent behavior toward the alteration of the above-mentioned parameters can be utilized as sensors, actuators, and drug delivery systems.^3^ One of the main groups of smart systems is magnetorheological (MR) materials. They can be classified into MR suspensions, MR elastomers, and MR gels.^23^

MR suspensions are systems consisting of magnetic particles (mainly carbonyl iron and its alloys) dispersed in a nonmagnetic fluid carrier medium such as mineral oil, silicone oil, or any other low-density synthetic oil.^24^ In the absence of a magnetic field, particles are randomly distributed in a carrier medium, and the MR suspension should ideally behave approximately as Newtonian fluids. When a magnetic field is applied, the dispersed particles create a chain-like structure along the lines of the magnetic field direction, which causes an increase in apparent viscosity of several orders of magnitude. In this situation, MR fluid can be considered as Bingham plastic material.^23,25^ The remarkable rheological changes, including a transition from a liquid-like state to a solid-like state, occur within microseconds and are reversible.^26,27^ The great controllability of the yield stress as a measure of the internal structure rigidity of MR fluids, dependent on the magnetic field strength, can be utilized in vibration control^28,29^ but also in many other applications, e.g., brakes,^30,31^ clutches,^32,33^ dynamometers,^34^ aircraft landing gears,^35^ and helicopter lag dampers.^36^

However, some drawbacks regarding MR suspensions limit their practical use, such as insufficient particles stability and their consequent sedimentation, poor oxidation stability resulting in thickening of the suspensions, and reduction of their magnetorheological performance.^24,27,37,38^ The density of particles is much higher than the density of carrier liquid; therefore, particles settle down under the action of gravity. Several approaches have been attempted to mitigate this drawback, e.g.: (a) covering the particles with organic or inorganic layer/layers to reduce bulk density and strengthen the interactions between the particles and carrier medium^39−47^; (b) the use of magnetic/nonmagnetic nanoparticles that act as a physical barrier preventing sedimentation^48−50^; and (c) addition of a special type of (hollow or porous) particles.^50,51^ Formation of inorganic or polymeric covering layer can also help in the improvement of oxidation stability of magnetic particles.^39−41^ Moreover, MR suspensions containing modified particles exhibit a lower coefficient of friction in comparison to MR suspension with neat particles; therefore, their usage allows for extending the service life of MR devices.^52^ Nevertheless, coating the magnetic particle surface with the nonmagnetic polymer can also cause a negative effect—leading to a decrease in saturation magnetization, when the polymer shell is synthesized in an uncontrolled manner.^53^

Some coating methods are utilized to obtain a polymer layer on magnetic particles, such as surface-initiated atom transfer radical polymerization (SI-ATRP),^39−41,54−57^ oxidative polymerization,^46,58,59^ and dispersion polymerization.^47,60−62^ SI-ATRP is one of the most important techniques for forming polymer layers on the surface of particles used in MR fluids since it enables to control polymer coating process.^39−41,54−57^ It belongs to the reversible-deactivation radical polymerization group and its main advantage is the ability to control polymer grafting density, chain length, composition, architecture, and functionality.^63,64^ Tuning grafting density and length of polymer chains allows for tailoring the thickness of the polymer layer created on the particle surface and consequently for adjusting the properties of particles. Cvek et al.^39^ reported that poly(glycidyl methacrylate) (PGMA) grafted on carbonyl iron (CI) particles via SI-ATRP significantly improved thermo-oxidation stability of CI particles as well as the stability of silicone oil suspensions, containing these particles, while almost did not affect the magnetic properties. Moreover, the authors showed that the molecular weight of polymer chains does not play a significant role in the improvement of MR fluid stability.^27^ This group^54^ also proved that the presence of PGMA chains on CI particles provides excellent antiacid corrosion properties. Similarly, as in the case of forming PGMA coating on CI particles,^39,54^ modification of CI particles surface with short poly(butyl acrylate) (PBA) chains via SI-ATRP improved chemical and sedimentation stability significantly, while saturation magnetization decreased negligibly.^40^ CI particles modified with poly(2-fluorostyrene), using SI-ATRP, were also utilized to prepare thermo-oxidatively stable MR fluids.^41^

To the best of our knowledge, there is no report about the application of CI particles, modified with dendritic polymers via SI-ATRP, in MR fluids. There are reports about CI particles functionalized with polyamidoamine (PAMAM) dendrons of generation 1.5,^55^ 2, and 2.5^56^; however, described CI particles were not modified using SI-ATRP in a controlled manner. First, acid-activated CI particles were functionalized with (3-aminopropyl)triethoxysilane (APTES) to introduce amine groups on the particle surface. Then, the growth of PAMAM dendrons was realized by iterative: (a) Michael addition reaction between the amine-terminated surface and methyl acrylate, resulting in the ester-terminated outer layer and (b) amidation reaction of the ester-terminated surface with ethylenediamine, resulting in new amino-terminated surface.^55,56^ PAMAM-modified CI particles exhibited improved thermal stability^56^ and oxidation stability in an acidic solution,^55,56^ in comparison to neat CI particles, while their saturation magnetization was only slightly lower than the saturation magnetization of unfunctionalized CI particles.^55,56^ Saturation magnetization of CI particles modified with dendrons of generation 1.5,^55^ 2,^56^ and 2.5^56^ decreased by 1.7,^55^ 4.5,^56^ and 4.7%^56^ in comparison to neat CI particles. PAMAM-modified CI particles were employed as a dispersed phase in MR suspensions. Functionalization of CI particles with PAMAM reduced the size of particle agglomerates formed in silicone oil and improved sedimentation stability.^56^

Therefore, this study is primarily focused on the fabrication of the CI hybrids with dendrite polymer structure on the surface of particles, prepared by SI-ATRP of poly(2-(trimethylsilyloxy)ethyl methacrylate). According to our best knowledge, such a type of material was not used for the overall improvement of CI particles as well as MR suspensions stability, while the MR performance was sustained on a similar level. The controlled manner of the dendritic structure fabrication was confirmed using proton nuclear magnetic resonance spectroscopy (^1^H NMR) and gel permeation chromatography (GPC). The significantly changed surface properties of CI-dendrites in comparison to simple brush structure were investigated using a scanning electron microscope equipped with an energy-dispersive spectroscope (SEM-EDS) and contact angle measurements. Significantly enhanced stability properties against corrosion as well as thermo-oxidation, sedimentation, and redispersibility were also confirmed, while the magnetization saturation decreased up to 5%. Finally, the magneto-responsive capabilities as well as long-term reproducibility were thoroughly investigated.

## Experimental Section

### Materials

*N*,*N*,*N*-Triethylamine (TEA, ≥ 99%), *N*,*N*,*N*′,*N*″,*N*″-pentamethyldiethylenetriamine (PMDETA, 99%), copper(I) bromide (CuBr, ≥ 99.999%), ethyl α-bromoisobutyrate (EBiB, 98%), α-bromoisobutyryl bromide (BiBB, 98%), 2-bromopropionitrile (BPN, 97%), methyl 2-bromopropionate (MBP, 98%), monomer 2-(trimethylsilyloxy)ethyl methacrylate (HEMATMS, 99%), *N*,*N*-dimethylformamide (DMF, 99%), anisole (99%), *N*,*N*-dimethylacetamide (DMAc, 99%), dimethyl sulfoxide (DMSO, 99.9%), anhydrous tetrahydrofuran (aTHF, 99.9%), acetone (99.5%), tetrahydrofuran (THF, 99%), isohexane (≥99%), 3-(aminopropyl)triethoxysilane (APTES, 97%), potassium fluoride (KF, 99%), and tetrabutylammonium fluoride (TBAF, 1.0 M in THF) were purchased from Aldrich (USA). HEMATMS monomer was purified by passing through a column filled with basic alumina before use. All other listed reagents were used as received. The CI particles (>97.8%) with average of 1 μm in diameter (ES grade, BASF, Germany) were used in this study. Silicone oil Lukosiol M200 (Koln, Czech Republic) was used as received.

### Synthesis Procedures

#### Modification of the CI Particles

The surface of the CI particles was activated by the treatment of the CI powder (100 g) with 0.5 M hydrochloric acid, and then the activated CI particles were functionalized with a silane agent according to the literature.^39^

### Synthesis of the CI-Brush Particles

The Schlenk flask containing the CI–Br particles (15 g) was evacuated and then backfilled with argon several times. The argon-purged HEMATMS (10 g, 49.4 mmol), EBiB (0.073 mL, 0.494 mmol), PMDETA (0.103 mL, 0.494 mmol), and anisole (10 mL) were added, and the mixture was degassed by several freeze–pump–thaw (F-P-T) cycles to eliminate the presence of oxygen. Finally, the flask was filled with argon, the CuBr catalyst (70.9 mg, 0.494 mmol) was quickly added to the frozen mixture under an argon flow, and an additional freeze–pump–thaw cycle was performed. The reactants were used at a molar ratio of [HEMATMS]:[EBiB]:[CuBr]:[CuBr_2_]:[PMDETA] = [1000]:[1]:[0.8]:[0.2]:[1.2], while anisole served as a solvent in the amount of 50 vol %.

**Figure 1:**
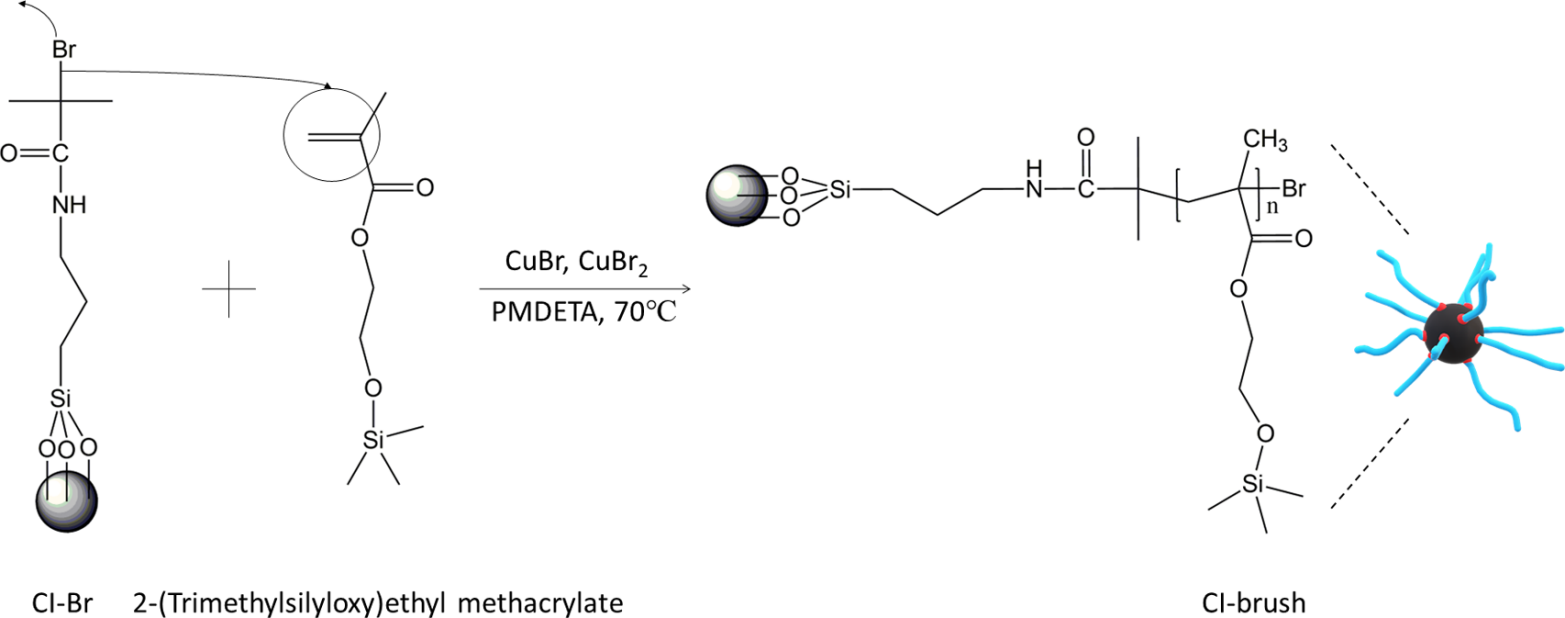
Schematic illustration of SI-ATRP of HEMATMS from the CI surface to obtain CI-brush particles.

To initiate the polymerization, the flask was immersed in a silicone oil bath preheated to 70 °C. The mixture was mechanically stirred in the compact glovebox (GP [Campus], Jacomex, France) under a nitrogen atmosphere (<10 ppm of O_2_). The reaction was stopped by exposure of the mixture to air and cooling to laboratory temperature. The prepared core–shell structures were purified by washing with THF (5 times, 100 mL each) and acetone (5 times, 100 mL each) using the accelerated decantation method with a magnet at the bottom of the beaker and then dried overnight at 60 °C under 200 mbar. The synthetic procedure is schematically shown in Figure 1.

### Synthesis of the CI-Dendrites

**Figure 2:**
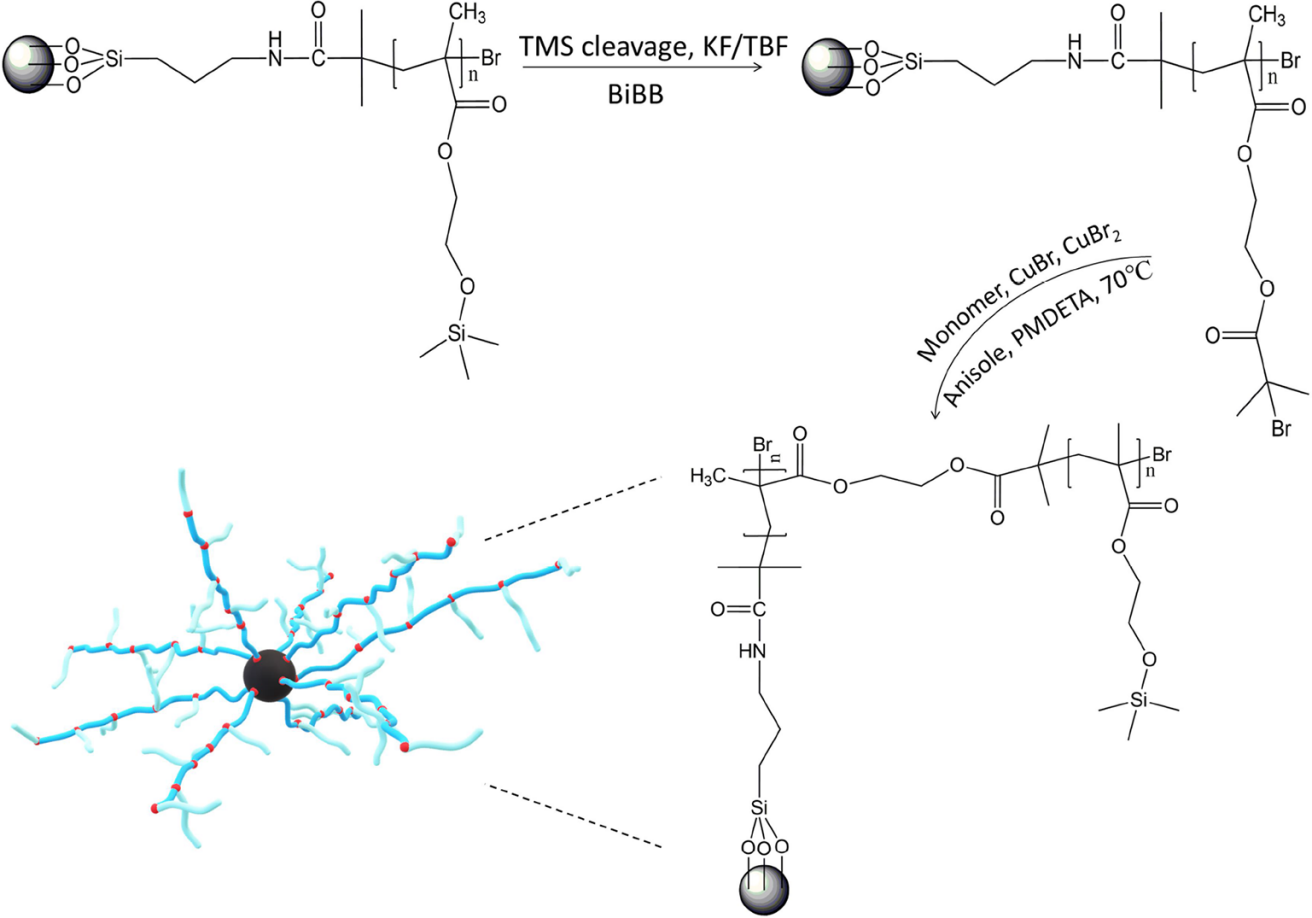
Synthetic procedure of the dendritic structure formation from the surface of CI-brushes through the CI-PBiBEMA midstep, followed by the synthesis of the CI-dendrites.

The Schlenk flask was equipped with 6 g of CI-PHEMATMS particles, and KF (2.2652 g, 38.988 mmol) and TBAF (0.23 mL, 1.0 M in THF, 0.229 mmol) were added directly to the flask with 40 mL of dry THF. The BiBB initiator (3.40 mL, 27.521 mmol) was added afterward dropwise with a volume rate of 10 mL h^–1^. The reaction mixture was stirred overnight. The excess of acid bromide was quenched by 1 mL of water and 1 mL of TEA. The solid product was collected by magnet and purified several times. The semiproduct CI-PBiBEMA (5g) was used for further extension of the CI-brush particles to obtain the CI-dendrites. In this procedure, CI-PBiBEMA (5g) was placed in an evacuated Schlenk flask backfilled with argon. Other components, such as HEMATMS, PMDETA, and anisole, were added and then four F-P-T cycles were performed. Finally, the flask was filled with argon, and CuBr and CuBr_2_ catalysts were quickly added to the frozen mixture under an argon flow, and an additional freeze–pump–thaw cycle was performed. The reactants were used at a molar ratio of [HEMATMS]:[CuBr]:[CuBr_2_]:[PMDETA] = [1000]:[0.8]:[0.2]:[1.2], while anisole served as a solvent in the amount of 50 vol %. The described procedure is schematically shown in Figure 2.

### Methods

Monomer conversion was determined by utilizing ^1^H NMR. The ^1^H NMR spectra were recorded on a Bruker Advance DPX 250 MHz (Bruker, Italy) instrument using deuterated chloroform (CDCl_3_) as the solvent. Number-average molecular weight (*M*_n_) and its distribution (*D*) were determined by using gel permeation chromatography (GPC). GPC measurements were performed with a Wyatt (Wyatt, Dernbach, Germany) instrument equipped with two perfect separation solution (PSS) columns and one guard column (GRAM Linear 10 μm, (*M*_n_ between 800 and 1,000,000)), differential refractometer (RI) and light scattering (LS) detectors. The measurements were performed using DMF as an eluent containing 50 mmol of LiBr, at a flow rate of 1 mL min^–1^. PMMA standards (*M*_n_ = 440 to 1,650,000 g mol^–1^) were used. The measurement temperature was 45 °C. For the characterization of functional groups of the samples, Fourier transform infrared spectroscopy (FTIR) was used. The measurements were conducted in the mid-infrared region of 4000–650 cm^–1^ with 32 scans using FTIR Nicolet 6700 spectrophotometer and OMNIC 3.2 software (Thermo Scientific Products: Riviera Beach, FL, USA). The ATR accessory equipped with a single reflection diamond ATR crystal on a ZnSe plate was used for all analyses. A scanning electron microscope (SEM, Tescan Vega II, Czech Republic) equipped with an EDS operating under an accelerating voltage of 5 kV was employed to provide information about the elemental composition of the studied samples on the surface of neat CI and variously modified CI using SI-ATRP. The densities of bare CI and CI-*g*-PHEMATMS particles were obtained using a gas pycnometer (UltraFoam 1200e, Quantachrome Instruments, Germany). The measurements were performed on dried samples at laboratory temperature, while nitrogen was used as a gas medium. The magnetic properties of bare CI particles, as well as their coated analogs (samples of approximately 150 mg), were investigated in an external magnetic field in the range of ±10 kOe (±780 kA m^–1^) using vibrating-sample magnetometry (VSM, Model 7404, Lake Shore, USA) under laboratory conditions. The amplitude of the vibrations was 1.5 mm, and the frequency was set to 82 Hz. To investigate the thermo-oxidation properties thermogravimetry (TGA) was used. Measurements were performed using a TGA/DSC1 analyzer (Mettler Toledo, Greifensee, Switzerland). Measurements were carried out in an oxygen atmosphere (flow rate 10 mL min^–1^), in the temperature range of 25–800 °C, with a heating rate of 10 °C min^–1^. The antiacid corrosion stability of bare CI particles and both types of CI-PHEMATMS particles were investigated. The amount of 1 g of appropriate particles was suspended in 20 mL of 0.05 M HCl, and the pH value was measured as a function of time. The instrument (SensoDirect pH110, Tintometer GmbH, Germany) was previously calibrated using two standard buffers at laboratory temperature. The acidic suspensions were mechanically stirred during the experiment, while before each measurement the probe of the pH meter was cleaned by rinsing with distilled water and drying. Sedimentation stability of the bare CI and CI-brushes and CI-dendrites-based suspensions was determined at room temperature by a UV–vis spectrometer (Shimadzu, Japan), using values of transmittance at 600 nm. Contact angle measurements were performed using silicone oil as a medium, and all investigations were done at room temperature. A defined volume (1.2 μL) of the silicone oil was deposited from a dosing syringe onto the surface of the sample (neat CO, CI-brushes, and CI-dendrites in the form of a pellet). Eight measurements were taken in different areas of each sample. The MR behavior of the prepared suspensions containing 60 wt % of the particles, in the absence as well as under various external magnetic fields, was studied with the help of an advanced rotational rheometer (Physica MCR502, Anton Paar GmbH, Austria) equipped with a magneto-device (Physica MRD 180/1T). The employed power supply provided an electric current of 0–3 A, which was correlated to the true magnetic field strength using a Teslameter (Magnet Physic, FH 51, Dr. Steingroever GmbH, Germany). The applied magnetic field was perpendicular to the MRE sample, which was placed between a parallel plate (PP20/MRD/TI) geometry and a measuring cell. The confirmation of the reproducibility of the MR phenomenon magnetic field on/off cycles was performed when alternating from 0 kA m^–1^ to 432 kA m^–1^ at a shear rate of 1 s^–1^, and in this respect, 20 cycles were performed. The off-state viscosity investigation for indication of the compatibility between the particles and silicone oil was performed using rotational rheometer Anton Paar (Physica MCR502, Anton Paar GmbH, Austria) equipped with a Peltier cell measuring cell. The investigated sample suspension was placed between a parallel plate (PP50, Anton Paar GmbH, Austria) geometry. All measurements were performed at a constant temperature of 25 °C.

## Results and Discussion

### Synthesis of CI-Brushes and CI-Dendrites

**Figure 3:**
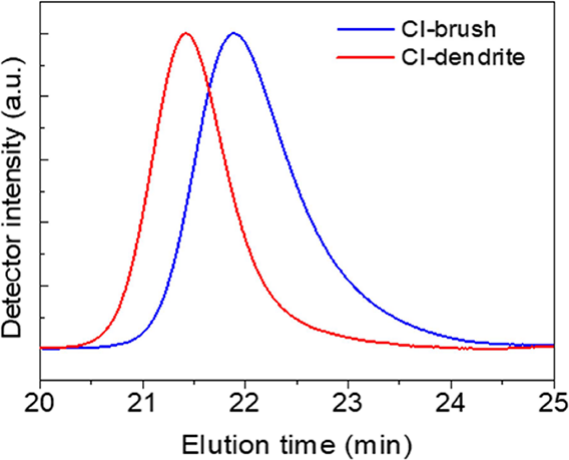
GPC traces for the CI-brushes and CI-dendrites.

The first step of the CI-dendrite fabrication was grafting of the CI surface with PHEMATMS brushes. This synthesis was performed according to Figure 1. The reaction was stopped after 3 h resulting in the brushes with molecular weight of *M*_n_ = 12,300 g mol^–1^ and *D* = 1.14. The monomer conversion calculated based on the ^1^H NMR was equal to 13%. The next step was the cleavage of the silyl moiety, followed by additional functionalization with the ATRP initiator, BiBB. Then, another reaction step under ATRP conditions, in which CI-brushes were grafted using the HEMATMS monomer, was performed. The whole procedure is schematically illustrated in Figure 2. The reaction was stopped after 6 h, with a monomer conversion of 9% and according to GPC, the final dendritic structure had a molecular weight of *M*_n_ = 21 200 g mol^–1^ and *D* = 1.28. The GPC traces are shown in Figure 3.

### Confirmation of the Coating of the CI Particles

**Figure 4:**
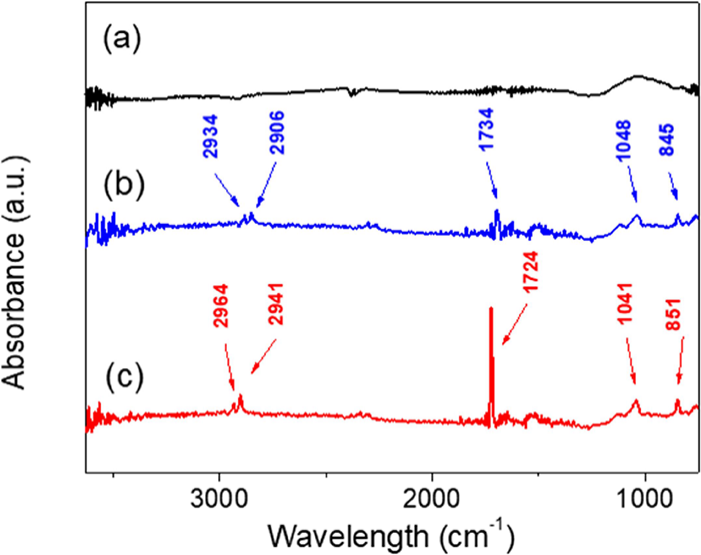
FTIR spectra of the neat CI particles (a), CI-brushes (b), and CI-dendrites (c).

The presence of the polymer brushes as well as dendrites on the surface of the CI particles was confirmed using various techniques, such as FTIR, SEM-EDS, and VSM. In the case of FTIR (Figure 4), the neat CI particles exhibited a typical spectrum without any significant absorption peaks similar as it was found elsewhere.^10^ The presence of the polymer brushes could be confirmed by specific absorption peaks at around 2934 and 2906 cm^–1^ corresponding to the CH stretching vibrations from the −CH_3_ and −CH_2_– groups. The peak at 1734 cm^–1^ occurring for CI-*g*-PHEMATMS particles could be attributed to C=O stretching vibrations. A raised absorption peak at 1048 cm^–1^ was assigned to Si–O–Si stretching, which is typical for organosilicon compounds. Finally, the peak at 845 cm^–1^ could be assigned to Si–CH_3_ rocking present in the pendant groups of the PHEMATMS, as already reported.^9^ In the case of the dendrites, the absorption peaks were very similar because the structure of the appended graft was the same as that of the initial backbone. Therefore, the peaks were slightly shifted, and their intensities were more pronounced. This effect was also because there was a higher amount of the polymer present on the surface of the CI particles.

**Figure 5:**
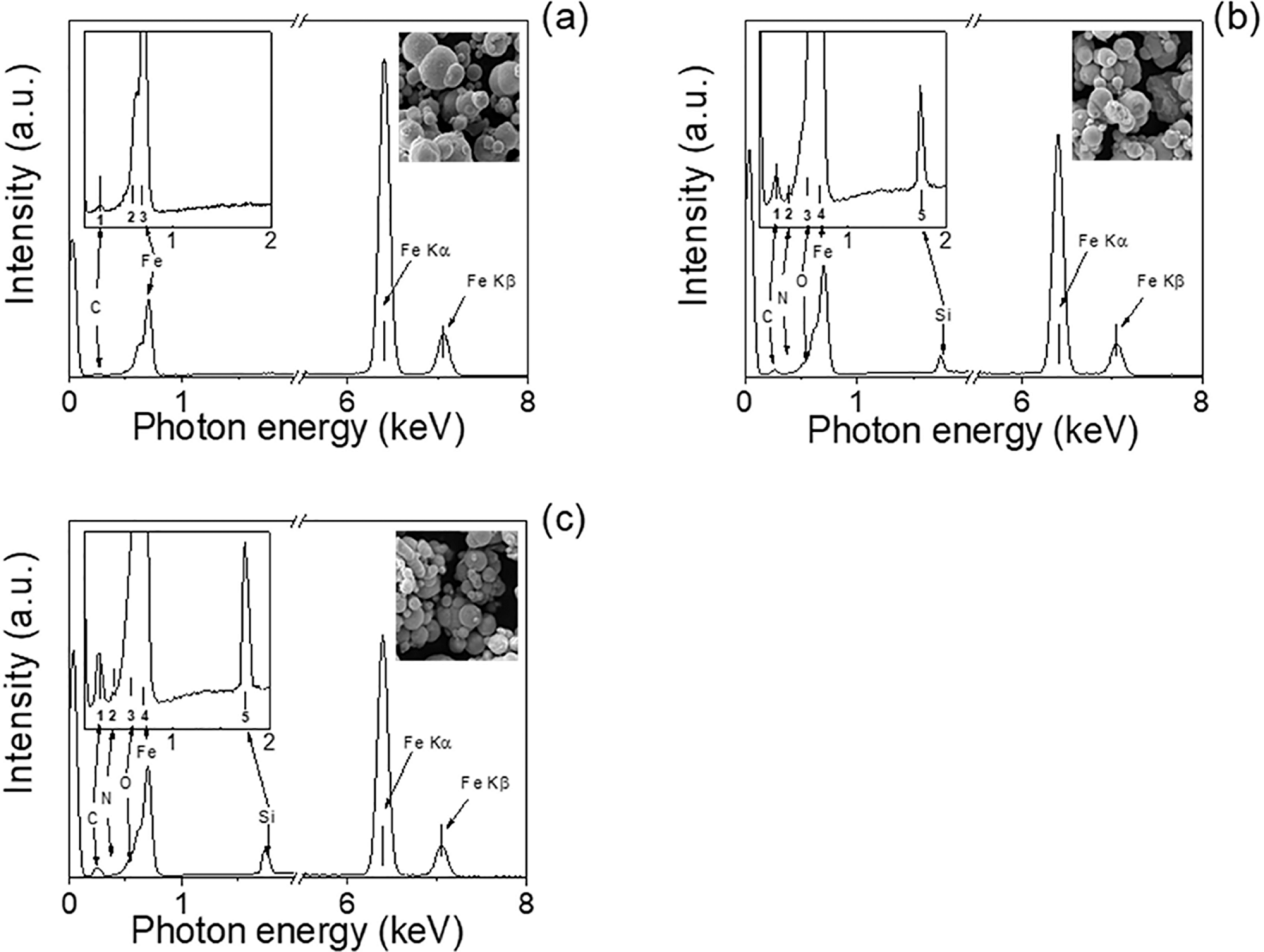
SEM-EDS spectra of the neat CI (a), CI-brushes (b), and CI-dendrites (c). The figure inset shows a higher magnification of the area from 0.2 to 2 keV. SEM images in all figures representing the area from the EDS data were collected.

CI particle surface was also characterized using SEM-EDS (Figure 5). In this respect, the neat CI particles showed typical spectra (Figure 5a) as published elsewhere.^65^ The presence of the polymer, PHEMATMS brush, was confirmed by an enhanced shoulder in the iron peak at low binding energies below 1 keV, corresponding to the higher amount of oxygen, due to the presence of the carbonyl functional group from PHEMATMS (Figure 5b). Another peak visible around 2 keV corresponds to the silicone. A similar spectrum was obtained for CI-dendritic structures; just the peak at 2 keV binding energy dedicated to the presence of silicone showed higher intensities (Figure 5c). Therefore, the successful coating of CI particles by PHEMATMS brushes as well as their dendritic graft structures was confirmed.

**Figure 6:**
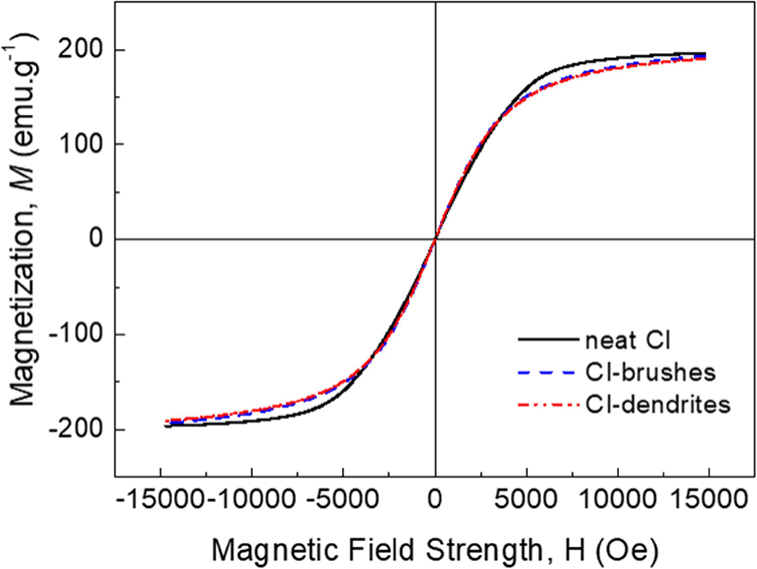
VSM spectra of the neat CI particles (black solid line), CI-brushes (blue dashed line), and CI-dendrites (red dash dot dot line).

A very important factor for the following application of the CI particles is the final magnetic activity of the CI-brushes and CI-dendrites. In this case, the neat CI particles showed a typical value of magnetization saturation around 197 emu g^–1^, clearly visible in Figure 6. The modification with brushes slightly decreased this value to 194 emu g^–1^, while dendritic structures slightly decreased it to 192 emu g^–1^. Moreover, the remnant magnetization as well as coercivity was influenced by such modifications (brushes or even dendrites) only marginally. Therefore, such hybrid particles were still considered to provide very promising magnetic characteristics for application in MR suspensions.

### Stability Properties

**Figure 7:**
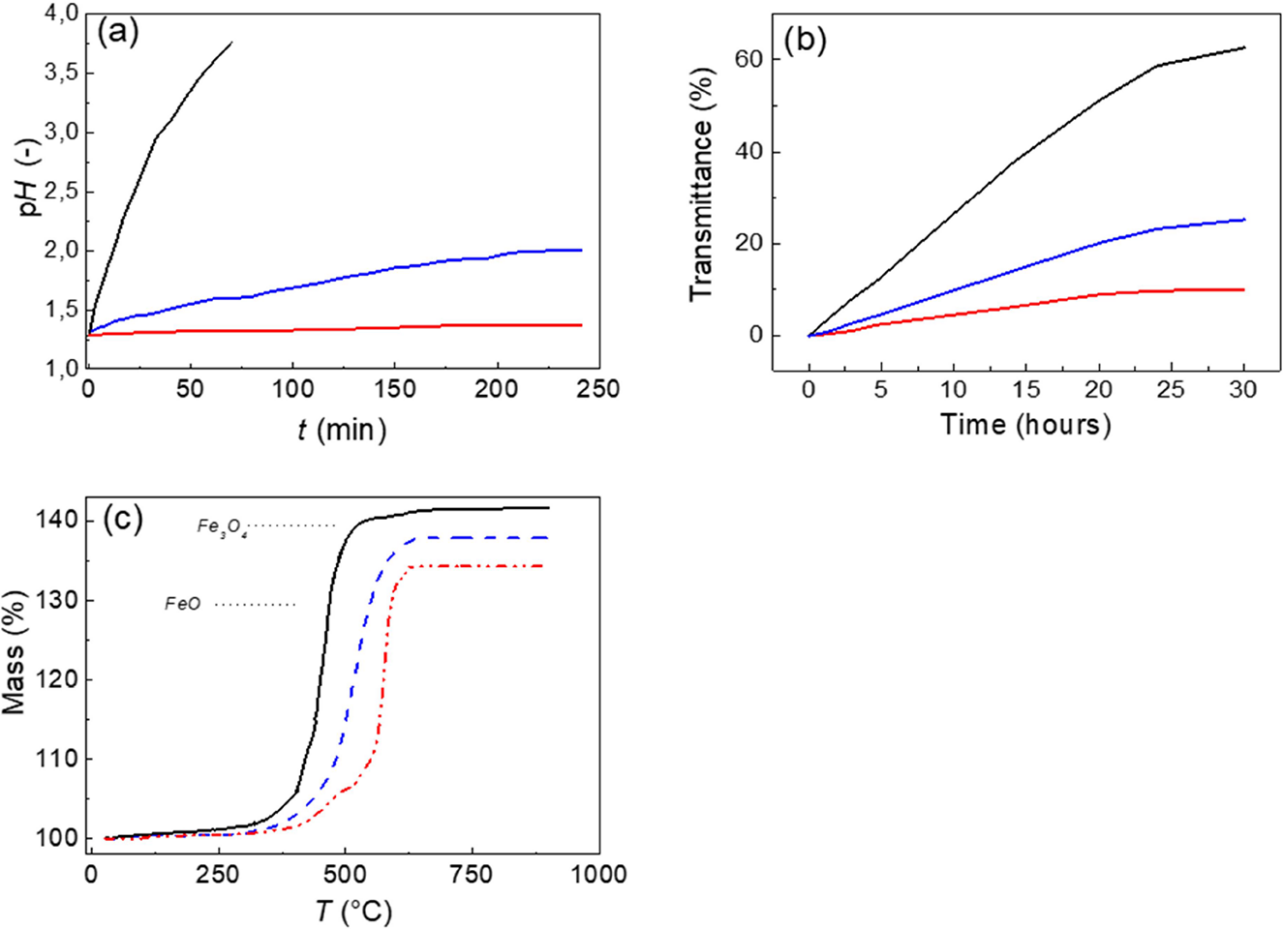
Dependence of the pH on the time duration during immersion in 0.1 M HCl of the various types of CI particles (a), sedimentation profiles investigated using a UV–vis spectrometer when transmittance against time is acquired (b), and thermo-oxidation stability as a change of mass against temperature is plotted (c), for neat CI particles (black solid line), CI-brushes (blue dashed line), and CI-dendrites (red dash dot dot line).

As already mentioned in the introduction part, the stability of the CI particles plays a crucial role in their final utilization in a smart application. Therefore, a set of stability tests (corrosion, thermo-oxidation, and sedimentation) were performed. In the former case, the corrosion stability is very important, and it was shown that the CI-brushes and CI-dendrites showed enhanced corrosion stability, which was significantly improved in comparison to the neat CI particles (Figure 7a). Moreover, the CI-dendritic particles showed significantly improved stability than those CI particles modified previously by our group using some organic moieties^66^ as well as other research groups.^53^ The CI-dendrite particles were stable for 6 h of the performed measurement in very concentrated hydrochloric acid.

Another very important factor that is often investigated in the case of MR suspensions is the sedimentation stability (Figure 7b). Here again, the neat CI-based suspension showed fast sedimentation since 60% of transmittance was reached within 30 h of investigation. The utilization of brushes improved this behavior significantly, while the CI-dendritic-based suspensions were very stable reaching less than 10% transmittance after 30 h and such values last for an additional 72 h. It must be pointed such values overcome the current state of the art published by Cvek et al.^57^

From the industrial applicability point of view, the thermo-oxidation stability needs to be investigated. The typical TGA measurement performed in air atmosphere was used (Figure 7c) for this purpose, as it was already commonly used by various research groups.^58^ The presence of brushes or dendrites on the surface of CI particles significantly shifted this stability toward higher temperatures of 50 or 120 °C, respectively. Therefore, it was concluded that the selected approach for particle modification significantly elevated their overall stability. Moreover, the dendritic structures could even enhance this stability behavior due to the more compact coating in comparison to the brush-type structures.

### Compatibility with Silicone Oil (Contact Angle and Off-State Viscosity)

**Figure 8:**
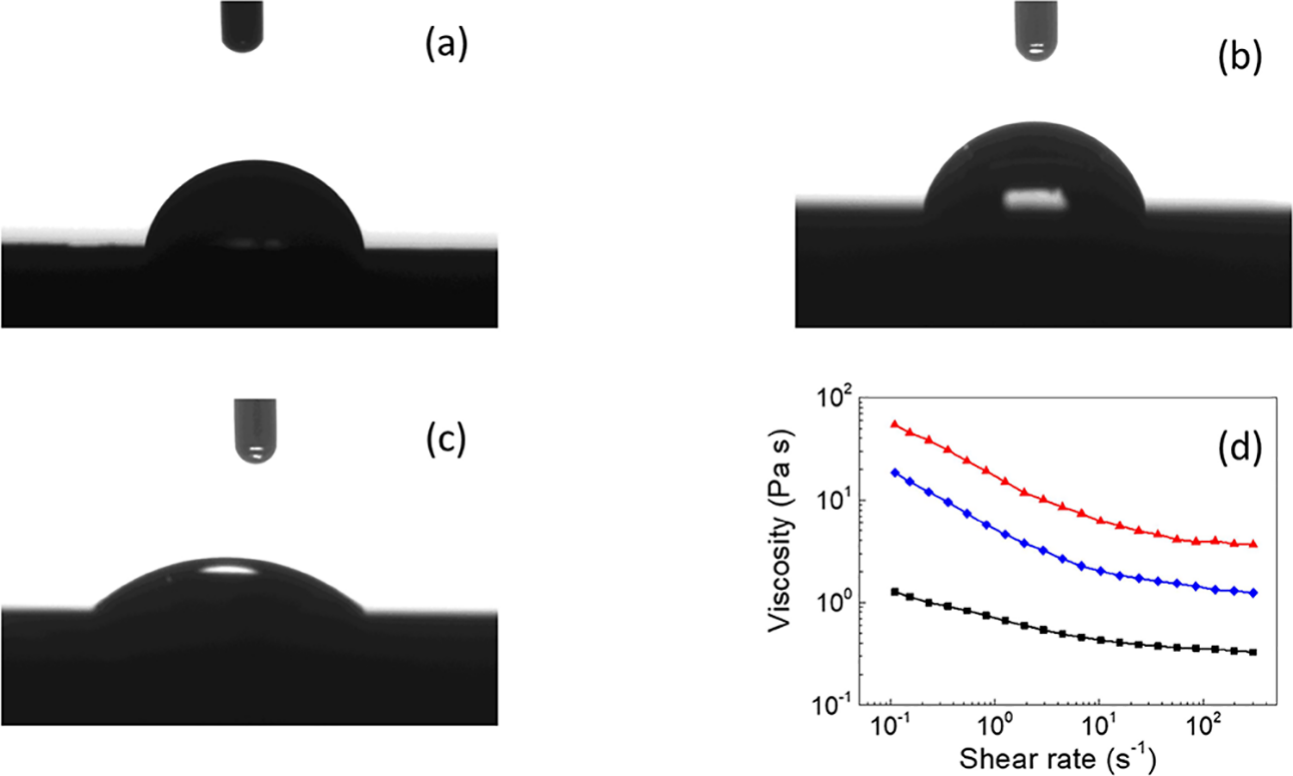
Contact angle measurements based on sessile drop, between the neat CI pellet and silicon oil (a), CI-brushes and silicone oil (b), and CI-dendrites and silicone oil (c) are presented. Dependence of the viscosity on the shear rate for various CI silicone oil-based suspensions, where neat CI (black square), CI-brushes (blue diamond), and CI-dendrites (red triangles) (d). All measurements were performed at room temperature.

The improved stability properties, especially sedimentation properties, are closely connected to the interfacial compatibility between the CI particles and silicone oil. In this respect, the contact angle between the neat CI particles, CI-brushes, and CI-dendrites and silicone oil was investigated (Figure 8a–c). It was found that the contact angle decreased after each step of the modification from 42.2° to 38.6° and 26.4°, respectively. Therefore, the tunability of the surface properties and thus the compatibility between the particles and silicone oil could be easily tailored. This is in close connection to the off-state values of the viscosity obtained from the steady shear rheological investigations (Figure 8d). The higher the viscosity of the suspension, the better the compatibility between the particles and medium.^67^ Thus, it was concluded that modification of the CI particles with PHEMATMS silyl-based methacrylate of various morphologies can be used as the specific approach for the synthesis of the designed coating, resulting in tunable surface properties.

### MR Performance (Steady Shear in the Absence and Presence of a Magnetic Field and Reproducibility of the Phenomenon)

**Figure 9:**
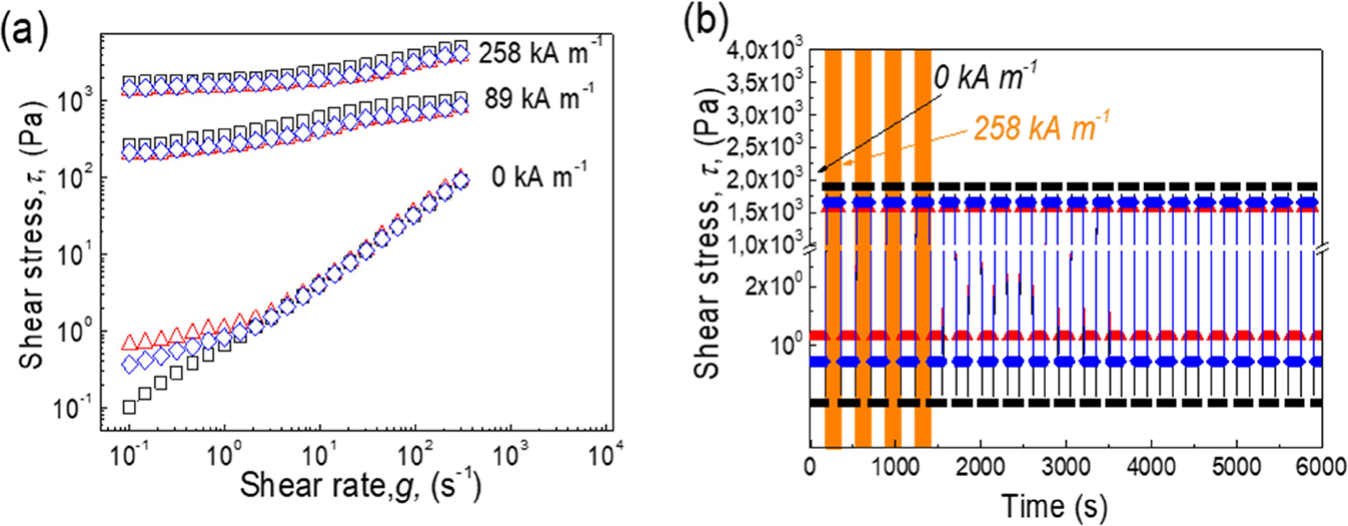
(a) Dependence of the shear stress on the shear rate at various intensities of magnetic field strength and (b) dependence of the shear stress on the time in *on*/*off* regimes of magnetic field, for neat CI (black square), CI-brushes (blue diamond), and CI-dendrites (red triangles). Orange fields are regimes in which a magnetic field of 258 kA m^–1^ was applied. Just four regimes are highlighted for better orientation in the figure.

Prepared suspensions showed very promising stability properties, and thus, the MR performance was thoroughly investigated. From Figure 9a, it can be seen that the rheological performance of investigated suspensions; in this case, shear stress can be tuned by the application of the external magnetic field, and subsequently change their behavior from nearly Newtonian to pseudoplastic, exhibiting a transition from liquid-like to solid-like state. Moreover, the decrease in MR performance was not dramatic since the magnetization saturation was decreased by the presented modification only negligibly. In this respect, the reversibility of smart MR capabilities is a crucial factor for the final application. Therefore, the cycling between switching *on*/*off* the magnetic field and fast response was monitored over 20 cycles (Figure 9b). It was found that all performed modifications did not significantly influence the reproducibility; however, they slightly influenced the *off-*state and *on-*state rheological behavior since *off-*state viscosity increased with modification while *on-*state decreased. It has to be mentioned that all obtained results confirmed very promising values of the yield stress which was found to be 1500 Pa for CI-brushes and 1300 Pa for CI-dendrites.

## Conclusions

In this work, both the CI-brush and CI-dendrite particles were successfully prepared using the SI-ATRP approach. Their fundamental characterization was performed using ^1^H NMR and GPC. The magnetic activity of these particles showed a very promising decrease of magnetization saturation up to 5% from the original values for neat CI. The successful modification was further confirmed using FTIR as well as SEM-EDS investigations. It was proved that such substantial structures significantly improved stability against corrosion and thermo-oxidation in comparison to the neat CI ones. The presented approach significantly changed the surface properties of the particles and thus improved the compatibility between CI and silicone oil, which consequently also improved sedimentation stability; such a CI-dendritic system was stable for almost 3 days. Finally, the elucidated MR performance was only negligibly affected for CI-brush and CI-dendrite-based suspensions with yield stress around 1500 and 1300 Pa, respectively. The obtained values are still very promising from the applicability point of view. Moreover, the reproducibility of the phenomenon was measured over 20 cycles and showed state-of-the-art MR performance since the alternating of the magnetic field was stable for all investigated samples, and dendritic structures present on the CI particles affected this capability negligibly.
